# The canine welfare, public health and environmental impact of systemic under-regulation within the UK puppy trade: A scoping review

**DOI:** 10.1017/awf.2025.10046

**Published:** 2025-11-04

**Authors:** Katharine Eloise Ross, Kirsten M. McMillan, Verity Bowell, Dylan Neil Clements, Stella Mazeri

**Affiliations:** 1 The Roslin Institute/The (Royal) Dick Vet School of Veterinary Studies, University of Edinburgh, Bush Estate, Midlothian EH25 9RG, UK; 2 Jeanne Marchig International Centre for Animal Welfare Education, Royal (Dick) School of Veterinary Studies, University of Edinburgh, Bush Estate, Midlothian EH25 9RG, UK; 3 Dogs Trust, 17 Wakley Street, London EC1V 7RQ, UK

**Keywords:** Animal welfare, canine welfare legislation, companion animal trade, one welfare, pet industry, public health

## Abstract

Almost a decade has passed since a DEFRA consultation concluded that existing legislation governing the UK puppy trade was “*outdated, inflexible, incompatible with current welfare legislation and cumbersome for both enforcers and businesses*”. The rapid outgrowth of the trade’s governing legislature, fuelled by contemporary consumer culture and the high degree of trader anonymity provided by the internet, has enabled a high-volume, untraceable and profit-driven market to evolve. Increased demand for puppies, exacerbated by social media trends and the COVID-19 pandemic, is sustained by an online medium that both encourages and capitalises upon modern-day ‘click-and-collect’ purchase behaviour. Moreover, the internet has only intensified the demand for pedigree and designer crossbreeds, many of which are shown to suffer lifelong physiological disorders caused by the positive phenotyping selection necessary to achieve breed standards. These factors have made puppies an attractively lucrative, low-risk commodity. Evidence of multi-level fraud and organised crime involvement has been revealed along the supply chain, resulting in systemic canine health and welfare issues. Whilst large-scale breeding operations reportedly smuggle unvaccinated puppies onto the British market from endemic (rabies, Leishmania) countries, high densities of pet dogs in urban areas is reportedly leaving high faecal-saturation levels, spreading anthelmic- and antibiotic-resistant pathogens. Meanwhile, unsafe concentrations of ectoparasiticides are detected in rivers and lakes. This review collates evidence from available sources that illustrate the current nature and impact of inadequate regulation in the UK puppy trade, aiming to support stakeholders in their efforts for essential and comprehensive regulatory reform.

## Introduction

The UK puppy trade has moved almost entirely online within the last two decades (Ares *et al.* 2021), evolving as a poorly regulated, largely anonymous, and profit-driven market with little traceability (Maher 2017). As the demand for puppies has consistently increased, accelerated by the 10-week UK lockdown during the COVID-19 pandemic, so too have the number and popularity of online sales channels that align with contemporary consumer preferences for immediate, ‘click-and-collect’ purchasing behaviour (Siettou 2021). Demand for pedigree and designer crossbreeds has raised the commercial value of the trade, whilst a shift from traditional to online marketplaces has introduced challenges relating to seller anonymity and traceability. This would appear to have attracted unethical traders who view puppies as high-value, cash commodities with comparatively low risk ((Muldoon *et al.* 2017). Instances of multi-level fraud, criminal activity, and concerns regarding canine welfare have been reported throughout the supply chain by government bodies, veterinarians and animal welfare organisations (Dogs Trust 2015; Four Paws International 2019; BVA & BSAVA 2020; Maher & Wyatt 2021). In 2016, DEFRA characterised the legislation governing the UK puppy trade as “*outdated, inflexible, and inconsistent with current welfare regulations*”, as well as burdensome for enforcement and businesses (Maher 2017). Eight years on, the Environment, Food and Rural Affairs Committee (EFRA)’s Pet Welfare and Abuse Inquiry was still calling for more up-to-date regulation, noting that enforcement was consistently hindered by underfunded local authorities facing capacity shortages and inconsistent training, leading to uneven application of animal welfare laws (EFRA Committee 2024). The inquiry also stressed urgent action on pet importation, improved biosecurity measures and transferring responsibility for border checks from carriers to government officials. The Licensing of Activities Involving Animals Regulations (LAIAR) in England (2018) and Scotland (2021) aimed to address these issues by strengthening breeder licensing, improving enforcement, and enhancing welfare standards across animal activities (UK Government 2018, 2021). However, a critical lack of empirical evidence on the trade makes any impact difficult to quantify (Ross *et al.* 2023). This scoping review collates the evidence that *is* available, to provide an overview of the current state of the UK puppy trade and potential legacy affects, highlighting where the gaps in legislation, regulation and enforcement may provide opportunities for exploitation, to the detriment of canine welfare, public health and the environment.

## Materials and methods

### Ethical considerations

On conducting this scoping review, ethical considerations included maintaining transparency and integrity in the selection and analysis of sources. Funded by the Dogs Trust, the review acknowledges an interest in advancing effective canine welfare legislation. To this end, it was essential to avoid any bias and base the review primarily on evidence available, as per Munn *et al.* (2018). Additionally, careful citation of sources was upheld to maintain ethical standards throughout.

### Study approach

This scoping review employed a hierarchical approach to examine the impact of current legislation and regulation of the UK puppy trade on animal welfare, public health, and environmental outcomes.

The existing statutory framework and enforcement mechanisms regulating the trade were reviewed first, followed by draft legislation and related government-issued guidance. Government agency reports, parliamentary committee reviews and inquiries were incorporated to provide evaluative perspectives from legislative and advisory bodies on the efficacy of existing measures and emerging concerns.

Non-peer-reviewed ‘grey’ literature (including reputable journalistic sources, reports from non-governmental organisations (NGOs), and conference proceedings) was also reviewed to provide additional perspectives. The use of these sources was undertaken sparingly and only incorporated when relevant information was unavailable from peer-reviewed or official sources. It is important to note the scarcity of relevant empirical data necessitated the inclusion of such ‘grey’ literature to ensure a more comprehensive understanding of the subject matter.

Finally, peer-reviewed journal articles were reviewed to gather empirical insights into the impact of the elements of the trade recorded in the reviewed literature.

## Introduction: The nature and scope of the UK puppy trade

**Figure 1.**
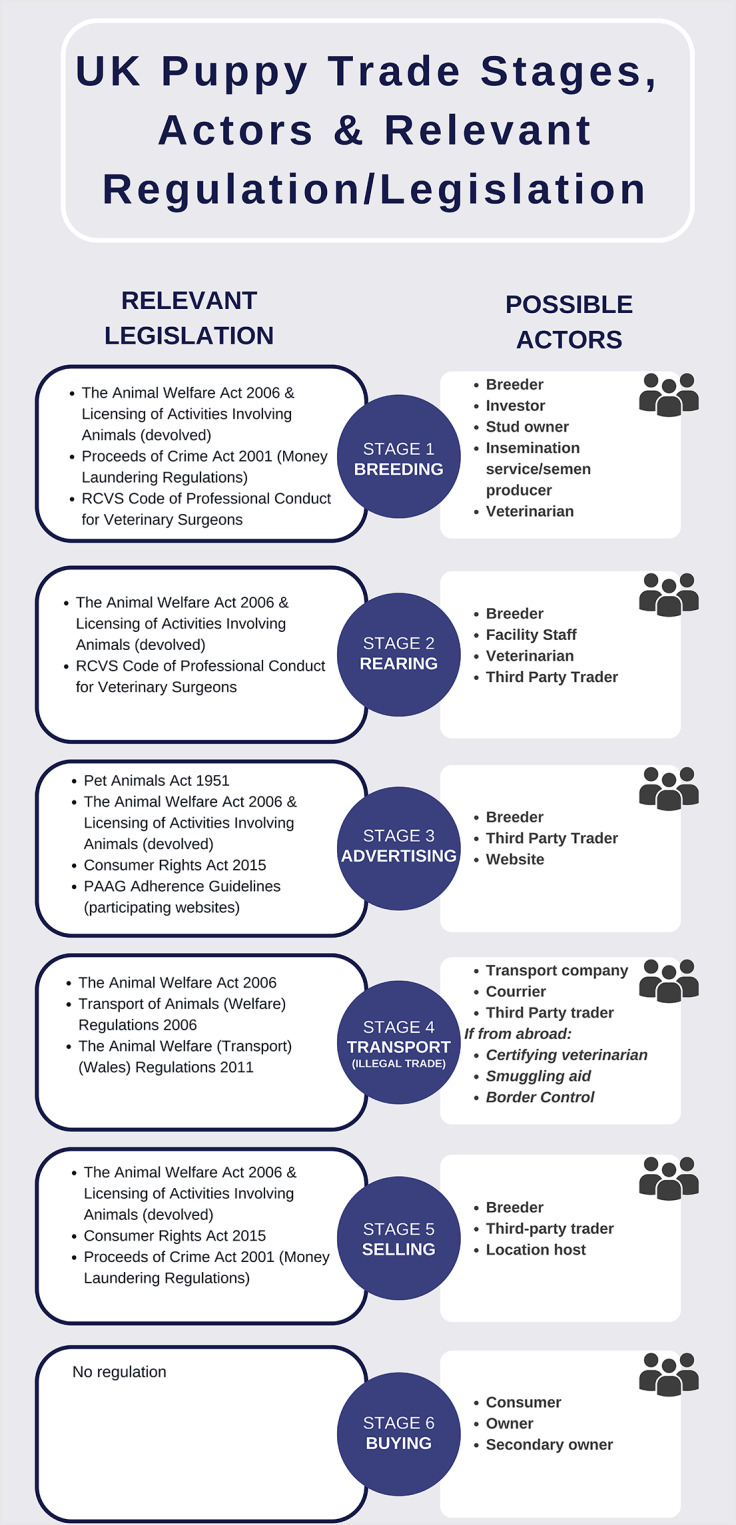
The chain of actions comprising the trade of puppies from production to purchase, annotated with potential actors and relevant legislation/regulation. It is important to note that progression of puppies through these stages is not necessarily linear. Some consumers may abandon the purchase process, and certain puppies may be culled or otherwise disposed of by breeders if unsold, and others may be returned to the breeder after purchase.

The UK puppy trade’s supply chain is inherently difficult to classify as ‘legal regulated’, ‘legal unregulated’ or ‘illegal markets’ (Maher 2017). These sequential stages typically flow from production to sale, each involving the contributions of various individuals who may act independently or in collaboration (see Figure 1) (Maher & Wyatt 2021). Meanwhile, and in contrast to other animal production industries, such as those involving farm or laboratory animals, the puppy ‘product’ remains largely untraceable (Four Paws International 2019).

These factors result in an overlap among the legal, illegal, regulated and unregulated markets, making it difficult for both consumers and regulators to distinguish between them. Figure 1 presents the chain of actions and potential actors involved, drawing upon stakeholder reports (Dogs Trust 2014; Four Paws International 2019) and Dr Jennifer Maher’s report on the connections between organised crime and the illegal trade of pets in Europe (Maher & Wyatt 2021). Throughout this chain, various actors may contribute to both short- and long-term health and welfare issues for dogs and puppies (Boyden *et al.* 2018), whilst also posing potential risks to public health (Trading Standards UK 2023). The reasons and motivations for this will differ, however. Some actors may contribute inadvertently, due to a lack of knowledge or being defrauded. Others may profit from these risks, causing harm through deliberate negligence and, potentially, malice. Recognising the complexity of these motivations and their underlying causes is crucial in order to inform the legislative requirements necessary for long-term change.

Either way, under current legislation, acting unethically or carelessly, does not necessarily mean acting illegally as even licensed, law-abiding and Kennel Club (KC)-registered breeders may be breeding dogs within preserved bloodlines or to aesthetic breed standards, practices that have well-documented links to long-term illness, disease, distress and premature death (Asher *et al.* 2009; Summers *et al.* 2010; Packer *et al.* 2013, 2015a; Packer & Tivers 2015; McMillan *et al*. 2024; The UK Kennel Club 2025).

Whilst some puppy breeders may well be acting completely lawfully *and* with a vested interest in animal welfare (Bateson 2010), it could be theorised that their overheads may be amplified by the provision of more adequate care, as compared to more profit-driven competitors. In fact, a number of dog welfare ethicists have debated the inherent ethical validity of breeding puppies at all, but certainly to aesthetic or pedigree preference, whilst so many dogs remain ownerless in shelter kennels (Bovenkerk & Nijland 2017; Menor-Campos 2024). As immediacy is increasingly favoured on online marketplaces by impulsive, and possibly emotionally driven target consumers (Iyer *et al.* 2020), it may logically follow that traders who produce puppies on a large scale, meeting the demand for instant availability and popular breeds, hold a larger proportion of the market compared to conscientious breeders. While there is a standard threshold distinguishing large-scale from small-scale breeders, such operations are typically characterised by a profit-driven volume of breeding activity with the emphasis on financial efficiency and output (Maher 2017; CARIAD 2021); this, at the expense of animal care, hygiene and waste disposal. Reports from these operations, dubbed ‘puppy farms’, have documented facilities housing anywhere from as few as five to over fifty breeding dogs (in addition to their litters), suggesting that scale alone does not determine classification, but rather a profit-driven operational intent and associated welfare conditions (Four Paws & Eurogroup for Animals 2018; Royal Society for the Protection of Cruelty to Animals [RSPCA] 2025).

Current regulations foster widespread misconceptions, as many definitions in the regulatory guidance materials regarding what constitutes a ‘legal’ versus an ‘illegal’ breeder appear unclear. The use of the self-coined term ‘hobby breeders’ is unofficial and contentious, as it tends to downplay the significance and seriousness of the animal welfare issues that can be associated with such breeding practices (EFRA Committee 2024). For instance, as shown in Table 1, ‘hobby’ breeders will not be considered a ‘business’ if they do not generate a profit (Canine & Feline Sector Group 2018); however, the definitions of ‘profit’ and ‘business’ appear ambiguous. While activities generating over £1,000 may qualify as a business for tax purposes, this depends heavily on their nature, frequency, and the breeder’s willingness to accurately report their earnings.

**Table 1.**
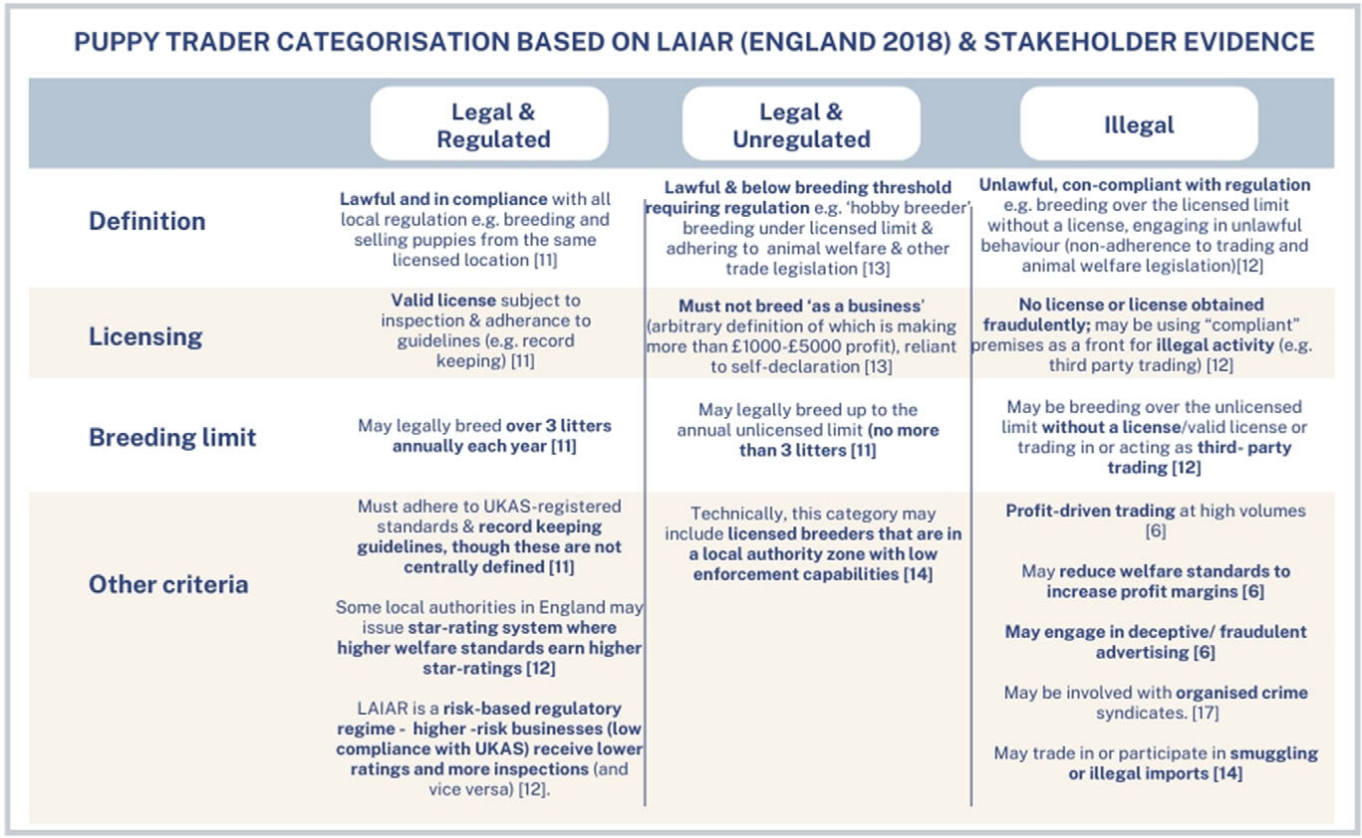
Categorisation of puppy traders based on breeding legislation covering England, with reference to further guidance materials on the regulations supplied by The Department of Environmental and Rural Affairs (DEFRA) and criminologist Jennifer Maher’s expert evidence on the illegal puppy trade.

Licensed breeding businesses are expected to be defined by standardised profit margins (UK Government 2024a); at the time of writing, the definition of these margins is not readily available on the KC website (The Kennel Club 2018b), leading to a lack of transparency and making it difficult for stakeholders to assess compliance. Furthermore, the trade is known to be characterised as a ‘cash’ or ‘spot’ market (Muldoon *et al*. 2017), indicating that transactions are primarily conducted in cash, on hand-over. This practice complicates efforts to track the sourcing of puppies and the identities of actors, resulting in minimal to non-existent audit or identity trails. The payment of deposits, which could offer a degree of traceability, has been deterred due to reports of deposit scams in which fraudulent traders request a deposit without any intention of selling the puppy (Pets Advertising Advisory Group [PAAG] 2020b).

Subsequently, it is difficult to track, trace or penalise non-conforming breeders or traders skirting existing laws. Even when illegal activities are identified and successfully prosecuted, penalties remain low (Maher 2017). Offenders face only short custodial sentences (up to 51 weeks), animal confiscation, non-permanent ownership bans, and on-the-spot fines limited to £5,000 (Maher 2017). A study of classified advertisements for puppies indicated that these fines may be less than the average advertised price for two French Bulldog puppies in 2021(Ross *et al*. 2023). For reference, the average litter size for French bulldogs has been reported at 4-6 puppies (Borge *et al.* 2011). Additionally, there is no system in place to prevent individuals that have been legally banned from owning animals from purchasing more dogs and breeding them for sale. Nor is there a system that would alert a buyer from purchasing a dog from such an individual.

Paradoxically it is the British public’s love of dogs that lies at the core of the issue: as the consumer value of puppies grows, so too does their value to profit-driven traders. The escalating commodification of dogs, in conjunction with inadequate regulatory frameworks and legislative oversight, has not only intensified adverse effects on canine welfare but rendered the trade increasingly susceptible to exploitation by organised criminal networks, thereby expanding broader negative consequences for society (Maher & Wyatt 2021). Amidst the challenges associated with defining what constitutes illegal or unethical practices within the trade, Yeates and Bowles (2017) successfully employed ‘rational choice theory’ to, initially, illustrate how the foundations of the trade may facilitate illegality. Rational choice theory, often utilised by criminologists in the study of organised crime, suggests that potential offenders engage in a logical thought process that consciously evaluates the elements of a target trade, while weighing the associated benefits, costs and risks. When the perceived benefits of committing a crime outweigh the costs or risks, individuals are more likely to engage in offending behaviour. Three of the theory’s key facilitating factors are relevant to the puppy trade, namely:

1. A lack of guardianship for both consumer and puppy due to inadequate legislation.
2. The presence of easily victimised, poorly educated and emotionally vulnerable or impulsive consumers.
3. A motivation for financial gain.

According to Maher (2017), these factors have been reportedly exacerbated by the movement of the trade online by providing anonymity, broad reach, and both streamlined advertisement platforms and advantageously opaque avenues for trading puppies.

### The online trade for puppies

An online marketplace is defined as a website or application (app) that connects sellers to interested buyers, aiming to facilitate a sale (Wang & Archer 2007). According to the PAAG, the majority of the online trade for puppies currently takes place via classified advertisement websites and social media apps (PAAG 2016). Puppies are marketed and advertised online, with verbal ‘orders’ placed through contact with the advertisers, but the actual exchange of money and puppy ‘product’ is conducted latterly, off-line. This means the website owners are separated from the supply and do not manage transactions (Wang & Archer 2007).

A scoping review, funded by the Scottish Government, found that these online media may directly facilitate the trade’s impact on animal, human, economic and environmental health (Maher 2017). The review concluded that the move online enabled the rapid evolution of the trade, allowing it to outpace existing legislation and regulation designed for a previously small-scale, offline market. This has led to increased challenges in protecting both animals and consumers from adverse effects associated with the trade.

The lawful advertising of all goods, including puppies, is defined by the Consumer Protection from Unfair Trading Regulations (UK Government 1968), under which it is specifically prohibited to:

- Use false or deceptive messaging;
- Leave out important information;
- Use aggressive sales techniques; and
- Design packaging deliberately to mislead consumers (Trade Descriptions Act 1968).

Reports by the RSPCA, the Scottish Society for the Protection and Care of Animals (SSPCA) and the Dogs Trust reveal that many online advertisements for puppies may often be engaging in all these practices (RSPCA 2016; SSPCA 2018). Neither proof of identity nor of residence is needed to advertise and sell puppies on the most popular classified advertisement websites and social media platforms. Indeed, emblematic ‘packaging’ or marketing of puppies is often characterised by the inclusion of idealised photos and descriptions in advertisements, which serve as visual and quality assurance information upon which many consumers base their purchasing decisions (Holland 2019). Although it is difficult to quantify the proportion of advertisements that are in breach of consumer protection laws, PAAG sets out guidelines for good pet advertising practice to safeguard welfare and consumer rights, and have revealed that during an analysis, 94% of social media advertisements for puppies did not meet their minimum guidelines (PAAG 2020b).

Various efforts have been made to educate consumers and formalise pre-purchase precautionary indicators to help them avoid illegal and/or unethical puppy traders. Unfortunately, these safety guidelines are often misappropriated as ‘playbooks’ by unscrupulous and/or fraudulent sellers. For example, recommendations from the multi-stakeholder-developed ‘Puppy Contract’ (AWF [Animal Welfare Foundation] *et al.* 2008) suggest that prospective buyers should see puppies with their mother as a marker of responsible breeding and to disprove suspicion of third-party trading. However, fraudulent traders have been reported as exploiting this advice by providing stand-in ‘mothers’ in photographs and at point-of-sale (Muldoon *et al*. 2017). The provision of a ‘mother’, fictional or otherwise, may offer a false sense of ‘safety’ to the transaction, without basis (Maher 2017). It is also reported that unethical traders import pregnant mothers into the UK under the Pet Travel Scheme (PETS), to produce puppies and be seen by buyers, before then returning them to their country of origin for re-impregnation (Dogs Trust 2014, 2015). To provide the illusion of ethical practice whilst maintaining anonymity, sellers may also employ tactics such as the use of aliases; renting temporary accommodation to present idealised breeding environments and/or to conceal true breeding facilities; and utilising disposable phones to sever contact once the puppy has been sold (SSPCA 2018). If signs of breeding malpractice are discovered post-purchase, these unethical breeders are, thus, untraceable. This is facilitated by the fragmented nature of the online puppy market, as the brief interval between the ‘click’, i.e. buyer contacting seller and agreeing to purchase, and the point of sale, ‘collect’, allows for the puppy to be relocated into a more appealing environment (EuroGroup for Animals 2023). This practice allows puppies to be transported in small numbers on-demand and aids in evading detection at borders. Concerningly, little information exists regarding the outcomes of overstocked puppies from high-volume breeding facilities that have outgrown their marketable age. What *is* known is that the well-documented practices of unethical trade will have short- and long-term effects on the welfare of both breeding stock and the puppies in question.

#### The impact of the illegal/unethical trade on canine welfare

A substantial body of grey literature, supported by a small, yet robust, scientific portfolio, provides insight into how canine welfare is impacted within all stages along the trade chain, starting at point of origin and continuing post-purchase. According to DEFRA (2018), a large proportion of puppies sold online are now suspected to originate from puppy farms. These breeding establishments are producing puppies for the main purpose of maximising profit, although definitive criteria for defining such operations are non-existent. Reports indicate that breeding stock and puppies often live full-time in poorly adapted wire cages, dirty whelping crates, or cramped runs, with minimal or no positive human interaction or enrichment (RSPCA 2025). Female breeding stock are bred frequently to maximise their worth and meet demand (RSPCA 2016), for which they are subject to mating at unhealthily young ages (e.g. at first season), as well as practices such as artificial insemination, biochemical manipulation of oestrus and restraint during/forced mating (Harney 2023). Veterinarians assessing seized breeding stock from puppy farms have documented cases of repeated Caesarean-sections in female dogs aged 2–8 years of age (Muldoon *et al*. 2017); and whilst canine oestrus can continue throughout a female dog’s life into her senior years (Yeates & Bowles 2017), no evidence could be found detailing the fate of such bitches once productivity declines.

Although provenance from a puppy farm may not be verifiable, veterinary professionals and NGOs have identified several associative, albeit non-definitive, indicators, based on raid, seizure and case study data. These markers include under-aged presentation, low weight at purchase, poor hygiene, dental disorder, parasitic infestation, and early signs of behavioural issues (British Veterinary Association 2016; Wauthier *et al.* 2018); all of which are linked to legacy health and welfare effects. These issues are attributed to the conditions found on puppy farms, as observed during raids, which include poor or inadequate housing and nutrition, high stocking densities, lax infection control, and failure to seek treatment or isolate individuals in the event of illness, along with unsuitable or delayed disposal of infectious waste or deceased animals (Maher 2017; Four Paws & Eurogroup for Animals 2018; CARIAD 2021; Brand *et al.* 2024).

**Figure 2.**
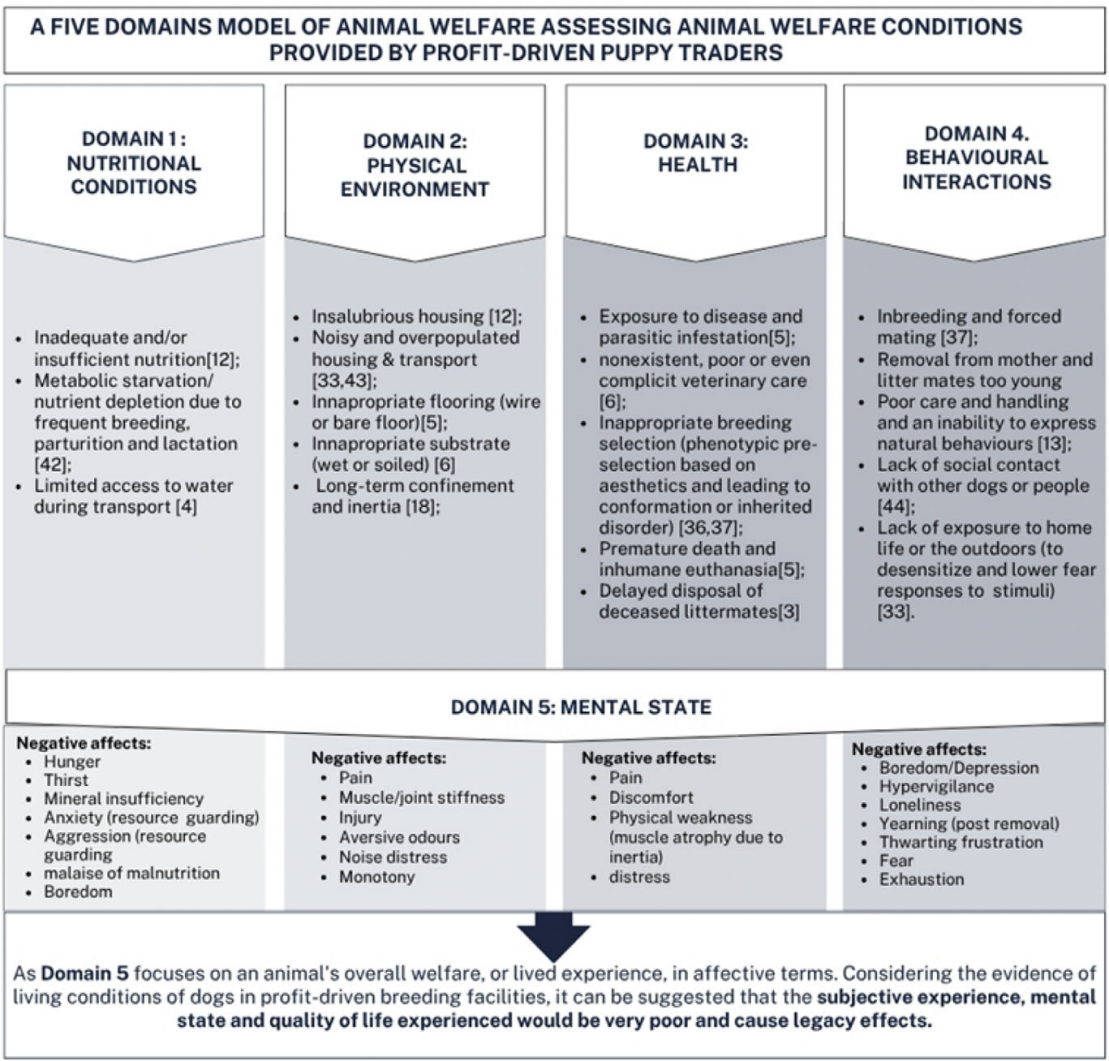
Evidence collated from animal welfare organisation puppy farm raid and seizure reports within the framework of Mellor’s Five Domains model (Mellor *et al.* 2020).

When considering the welfare of breeding stock and puppies pre-purchase, Mellor’s Five Domains model (Mellor *et al.* 2020) was employed where raid and seizure evidence offered a glimpse into the quality of life and affected mental state of these dogs (Figure 2). It is evident that the overall welfare of these dogs is very poor, many of them may live up to, or beyond, ten years as breeding stock within these facilities.

A survey undertaken by the KC for ‘Puppy Awareness Week 2018’, revealed that 25% of puppies bought online died prior to reaching six months of age while 18% were diagnosed with an illness requiring long-term veterinary treatment (The Kennel Club 2018a). A large-scale online survey in the US showed that ‘farmed’ puppies scored less favourably than puppies from small-scale breeders on eleven of the 14 sub-scales of the Mini Canine Behavioural Assessment and Research Questionnaire (mini C-BARQ) (Wauthier *et al.* 2018). Butler and Douglas (2016) reported that the canine study subjects in their study that came from commercial breeding establishments exhibited higher levels of behavioural problems, including increased aggression, fearfulness, and separation anxiety, compared to dogs from reputable breeders. Separation anxiety in dogs is characterised by behavioural indicators that include house soiling, destructive behaviours, persistent vocalisation, and aggression toward strangers, all of which have been linked to a heightened risk of euthanasia due to behavioural problems (Hitchcock *et al.* 2024). It is important to note that most studies linking unethical puppy trading practices directly to health and behavioural issues are based almost entirely upon owner or foster-carer reported data, which can be unreliable due to selection and response bias, notably as a result of the sensitive nature of the subject. This further highlights the lack of empirical data regarding the trade, as well as the lack of consumer accountability. It is also important to consider that, post-purchase, behavioural issues may develop due to the husbandry and care provided by potentially impulsive, poorly informed or adapted ownership styles.

### Consumer culture, popular breeds and conformation disorder

The dynamics underpinning the commercial puppy market are highly influential, albeit nuanced and under-investigated. There is evident public interest in acquiring puppies and aesthetic characteristics are known to play a significant role in purchasing decisions (Holland 2019). It is, however, debatable whether the market follows a purely demand-led model. Research has shown that markets reflect consumer interest in extreme phenotypes (Sandøe *et al.* 2017), but may also have their preferences shaped externally, by the availability and promotion of such dogs, or the influence of social media and/or cultural trends (Holland 2019; Pirrone 2020; Burnett *et al.* 2022). This raises questions regarding the extent to which demand is constructed by supply-side actors.

The increased popularity of online shopping, which has been seen to encourage impetuous ‘click-and-collect’ purchasing behaviour in other markets (Lidholm *et al.* 2017), has indeed made puppies rapidly accessible to impulsive, unvetted consumers who reportedly make choices based primarily on aesthetics, status features and trends (Sandøe *et al.* 2017). This is accentuated by the lack of accountability expected from consumers choosing to purchase a puppy. According to Packer *et al.* (2012), the multitude of harms caused by unethical trades in animals can be directly associated with consumer culture, most notably the normalisation and even veneration of health issues (e.g. snoring and snorting deemed ‘cute’ by brachycephalic owners) linked directly to extreme conformation (Packer *et al.* 2012).

When the regulatory amendment to the Licensing of Activities Involving Animals Regulations (LAIAR) (commonly referred to as ‘Lucy’s Law’) introduced a specific prohibition on licensed pet sellers selling puppies or kittens that they had not themselves bred, several outlets, including BSAVA News (BSAVA 2020) and even the UK Government (DEFRA 2020)website reported that henceforth, "…*anyone wanting to buy a puppy **must** now buy direct from a breeder, or consider adopting from a rescue centre*”. However, there is no provision for consumer penalty within the wording of the Act (UK Parliament 2019) and, in practice, consumer compliance relies largely on prior knowledge and individual diligence rather than enforceable legal obligation. It follows that unscrupulous sellers may also be capitalising on a free market where consumer ignorance and/or fashion driven/impulsive online purchasing behaviour is not only accepted and normalised as well as legally unaccounted for. An RSPCA survey (2012) reported that 40% of dog owners spent less than a week performing pre-purchase research. According to the 2016 People’s Dispensary for Sick Animals Animal Wellbeing (PAW) Report, approximately 5.2 million respondents, or 24%, reported not to have engaged in any research before acquiring their pet (PDSA 2016). Although this figure had reportedly decreased in 2020 to 19%, and again in 2024 to 13%, it indicates a significant proportion of the target market may be purchasing puppies with little to no knowledge of how to do so ethically and in accordance with animal welfare standards (PDSA 2020, 2024).

Meanwhile, although a survey-based study reported that 74% of first-time owner respondents claimed to undertake research, it averaged out at under a day of pre-purchase research (RSPCA & Pannett 2012).

This kind of consumer behaviour was further highlighted by a study of canine acquisition motivations during the COVD-19 pandemic which reported that, whilst an estimated 3.2 million dogs were acquired in the UK during lockdown, survey respondents appeared driven primarily by intrinsic motivations that served their own interests rather than the welfare of their prospective pets (Brand *et al.* 2022). These included encouragement to exercise, improvement to their family’s mental health and providing companionship to their children whilst they worked from home. A subsequent study by the Battersea Dog and Cat Home (2021) revealed that 19% of owners regretted their purchase of a dog or cat during the pandemic due to “*costs, demands on their time and the behaviour of their pet*”; 20% of these owners said they had not considered the long-term implications of acquiring a pet during lockdown and subsequently returning to work, and 15% admitted they had made a ‘mistake’ in purchasing a pandemic pet. Prior to the pandemic, an RSPCA report showed that 1 in 5 puppies bought online in Bristol were no longer with their owners two years following acquisition (University of Bristol 2011). In 2021, The Pet Food Manufacturers Association (PFMA 2021) reported a 12% pet relinquishment rate, suggesting an estimated ~450,000 pets had been relinquished over that year alone.

In response to consumer demand, online puppy traders also appear to favour popular breeds, including purebred dogs, bred to KC breed standards, or ‘designer’ crossbreeds (DCBs) (Burnett *et al.* 2022; Ross *et al.* 2023). Many of the UK’s most popular breeds are bred for aesthetic features that are the product of inbreeding and positive selection. A study of online classified advertisements selling puppies in the UK revealed that 66% of all adverts across three platforms were for the same 20 breeds, and 46.9% were for breeds linked to conformation disorders. Extensive evidence is available regarding the associations between positive selection to achieve breed standards and health and welfare issues, including: orthopaedic and joint disorders (LaFond *et al.* 2002; Demko & McLaughlin 2005); skin disease (Asher *et al.* 2009; Summers *et al.* 2010; Mazrier *et al.* 2016); aural disease (Hodgman 1963; Asher *et al.* 2009; Hayes *et al.* 2010) ocular disease (Asher *et al.* 2009; Collins *et al.* 2011); breathing difficulties (O’Neill *et al.* 2015; Packer & Tivers 2015; Packer *et al.* 2017); and spinal disorders (Ryan *et al.* 2017). The conformation of certain breeds (e.g. bulldogs) may make mating and parturition difficult or even impossible (Rooney & Sargan 2010; Borge *et al.* 2011). Repeated C-sections, human-facilitated breeding and unregulated artificial insemination practices are employed to breed these dogs (Muldoon *et al.* 2017). Studies have also demonstrated that common inbreeding practices, such as use of ‘popular sires’ and lineage conservation heavily diminish genetic diversity (Jansson & Laikre 2014), increasing genetic disease risk. A study by Packer *et al.* (2017) on owner’s perceptions of Brachycephalic Obstructive Airway Syndrome (BOAS) shows that severe symptoms, such as respiratory distress, exercise intolerance, upper respiratory noise and collapse are perceived as normal, healthy behaviours by 58% of those surveyed. These dogs have also been seen to suffer from limited communication, causing social difficulties and behavioural issues (Eretová *et al.* 2024). Although high-profile documentaries, such as the BBC’s *Pedigree Dogs Exposed* (2008) and *The Truth About Your Dog* (2019) were said to be ‘pivotal’ moments in the campaign to raise awareness of these issues, a study of the market between 2019 and 2020 revealed the majority of online adverts for puppies were still for breeds linked to extreme conformation or well-documented hereditary disorders (Poling & BBC Scotland 2015; Victory & BBC Three 2016; UNILAD 2018; Heaney 2019).

And yet, framing the market as being solely driven by consumer preferences risks obscuring the influential role that breeders and traders — particularly those producing and selling dogs with exaggerated or extreme conformations — play in creating and sustaining this demand. On the available evidence, it seems that a mix of trader and consumer factors are driving the market. Whilst existing legislation and regulation tends to codify and sanction the responsibilities and transgressions of breeders and traders alone, it fails to recognise those of consumers. This may be due to the recognition that many consumers are clearly being misled and often defrauded by puppy traders. Conversely, the press, as well as stakeholder interventions, appear to lay the blame for this with the consumers themselves. Nonetheless, the consequences of the trade are clearly far-reaching, impacting not only canine victims but also human stakeholders, including consumers and the broader public.

### The trade’s impact on the environment and public health

Although there is a paucity of reports dealing with the environmental impact of UK puppy farms, studies in the US have demonstrated the substantial environmental damage caused by improper waste disposal at large-scale breeding facilities, including dog faeces, food waste, soiled bedding and deceased animals (Towsey 2010; Gill 2013).

The nature of trade has enabled a surge in the canine population, particularly in urban areas, implying an increased concentration of urine and faeces in streets and parks (Mori *et al.* 2023). Dog and cat faeces are now recognised as reservoirs for novel antibiotic resistance genes and anthelmintic-resistant parasites (Cinquepalmi *et al.* 2012; Da Silva *et al.* 2021), some of which are zoonotic and capable of posing a potential risk to human health. Parasiticides that play an important role in flea and tick treatments administered to dogs are being found in lakes and rivers at concentrations that exceed safe limits for local wildlife (Traversa *et al.* 2014), whilst dog urine and faeces left in peri-urban green spaces and national parks have been shown to change soil composition thereby affecting flood and water logging as well as modifying ecosystems to the detriment of wildlife and biodiversity (Allen *et al.* 2020; De Frenne *et al.* 2022; Massetti *et al.* 2022). Meanwhile, reports of out-of-control dogs disturbing and killing sheep and wildlife have increased since the dog population’s sharp increase in 2020 (The National Sheep Association 2024; The Wildlife Trusts 2023).

**Figure 3.**
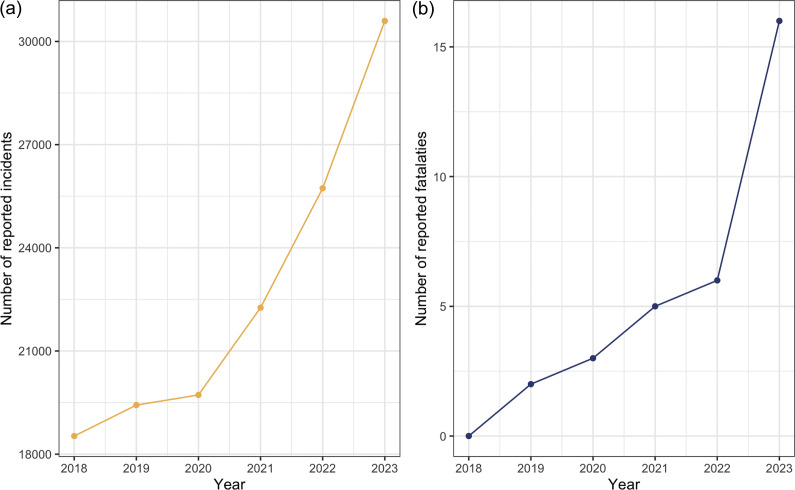
Annual total (a) dog attacks and (b) dog-bite fatalities reported by police in England and Wales between 2018 and 2023. Graphs based on Freedom of Information data, and no comparable data were accessible for Scotland or Northern Ireland.

According to data obtained by Freedom of Information (FOI), incidents of dog bites recorded by the police in England and Wales have increased by 34% between 2020 and 2023, and the increase in fatal attacks has more than doubled from 2020–2023 (Figure 3) (Office of National Statistics 2023).

Although correlation does not prove causation, the increased availability of dogs for purchase has indeed coincided, at least temporally, with reports that suggest a rising number of puppies being sold on the UK pet market may have experienced suboptimal husbandry and handling in their early years, and reports of a substantial proportion of the market purchasing on impulse, without prior research (Maher 2017; RSPCA & Pannett 2012). Simultaneously, several studies have reported increased prevalence and severity of conditions resulting from extreme phenotypic selection, such as exaggerated conformation or inherited diseases that may cause chronic pain and/or discomfort (Asher *et al*. 2009; Summers *et al*. 2010; McMillan *et al.* 2024). These factors have been associated with higher rates of human-directed aggression in dogs (Howell *et al.* 2025), implying a link between dog-bite incidents and unethical puppy trading. Another risk posed to public health by the illegal trade in dogs is the introduction and spread of endemic and potentially zoonotic disease (e.g. rabies and Leishmania) via the smuggling of unvaccinated and untraceable puppies from abroad for sale in the UK (Dogs Trust 2014, 2015; PAAG 2020a).

### Smuggling and illegal imports

The Dogs Trust have repeatedly demonstrated the ease with which puppies bred outside the UK were smuggled or illegally imported for sale (Dogs Trust 2014, 2015; BVA and BSAVA 2020), notably when they reported the successful smuggling of a fake dog into the UK in three out of four attempts due to inadequate document and identity verification (Dogs Trust 2015). Written evidence submitted to UK parliamentary committees in 2018 highlighted that between December 2015 and July 2018, Dogs Trust reported 1,433 intercepted puppies intercepted at UK borders, with 60% deemed illegally imported (Dogs Trust 2018).

Large-scale puppy traders smuggling puppies into the UK have been linked to organised crime syndicates (Maher & Wyatt 2021), working with well-remunerated veterinarians to provide certification and perform unethical procedures, such as breeding stock vocal chords severance (to avoid noise complaints), and mutilations banned in the UK on welfare grounds, including aesthetic tail docking and ear cropping (RSPCA 2021; Four Paws International 2024).

The 2021 Dogs Trust Puppy Pilot scheme, in support of the Animal and Plant Health Agency (APHA) in the interception, rehabilitation, and rehoming of illegally imported puppies at UK ports, reported an average age of seized puppies of ~8 weeks, 3 weeks younger than the 2019 average, and half the legal age of import (15 weeks) (APHA 2021). The youngest puppies were intercepted at just 4 weeks old, at which age a puppy’s contact with its mother and consumption of mother’s milk are highly important for its physical and psychological health (Scott 1958; Harvey *et al.* 2016). As previously mentioned, puppies may be imported illegally into the UK for sale smuggled within pregnant mothers imported as pets who, following parturition, are suspected to be returned to breeding establishments to be re-bred (Zucca 2021).

Contrary to The Welfare of Animals (Transport) Order (2006), transport can last up to 33 h, often originating from central/eastern Europe, without breaks for food, little to no water, or opportunity to exercise or toilet (Maher & Wyatt 2021). High stocking densities on these journeys increase the risk of diseases spread by faecal-oral transmission, such as Parvovirus (Mira *et al.* 2018). To avoid detection, smuggled puppies may be dowsed in oil (PAAG 2020a), or sedated (Four Paws International 2013). They may be transported under built-in ‘dummy’ boot floors and in the ceilings of ‘family’-style cars with reduced airflow and oxygen availability (Zucca 2021; Four Paws International 2022). Smuggled puppy seizure reports describe transport-related fatalities due to hypoxia and overheating/dehydration or pre-existing illness (Dogs Trust 2017). It was reported by Maher and Wyatt (2021), based on a body of evidence that included UK Border Force raid and seizure data, that organised crime involvement in puppy smuggling was highly likely.

### The puppy trade’s links to organised crime

During an EFRA enquiry into the international puppy trade, written evidence by criminologist, Dr Jennifer Maher, outlined ample evidence that significant portions of the UK puppy trade are being funded and are, in turn, *funding* organised crime, with law enforcement agencies recently coining puppies as the ‘new narcotics’ (Maher 2017).

In 2017, an APHA consultation as part of a Scottish government review of the illegal trade in puppies reported the “*sheer number of illegal puppies — one in every tenth car*” was said to be symptomatic of “*widespread and organised criminal network and activity*” (Maher 2017). During a 2018 Four Paws International virtual symposium on the trade, puppy trafficking was labelled the third largest illegal trade in Europe and aiding the financing of activities such as the trade of illicit drugs, weapons and human trafficking (Four Paws International 2018). These links have been corroborated by the Crown Office and Procurator Fiscal Service in 2019 who report evidence that puppies are used to finance gang crime in Scotland, corroborated by the SSPCA, who have labelled the unethical online trade in puppies a ‘black market’ (Maher 2017). In 2021, Her Majesty’s Revenue and Customs (HMRC) reported having recovered around £5 million unpaid taxes over four years via help from their ‘dedicated puppy task workforce’, with the UK puppy trade being worth anywhere between £100 million to £300 million annually, based on an estimated of 700,000 to 1.9 million puppies sold annually (Maher 2017). In 2019 and again in 2021, criminologist Jennifer Maher submitted evidence on the trade’s links to crime during an EFRA committee inquiry into the trade (Maher & Wyatt 2019, 2021).

Many of the stages of the puppy trade (Figure 1) require legitimisation via paperwork, documentation and adhering to ‘regulations’ set out by educational messaging, intervention, guidance or the law. Sophisticated and multi-player organised criminal gangs may exploit legislative loopholes and enforcement capabilities to manipulate or hide behind a ‘legal’ facade (Maher & Wyatt 2021). Forged documents can make the trader appear legitimate, misidentified by insufficient border control. Professionals, such as border officers, transport couriers and veterinarians may be criminal ancillaries or unwitting participants due to legislative and regulatory loopholes. A high-profile case even revealed a veterinarian inspector for the Department of Agriculture in the Republic of Ireland to own a puppy farming and trading operation (Four Paws International 2022). Covert investigations by the Dogs Trust have identified veterinarians in eastern European countries who accept remuneration to forge paperwork to facilitate the movement of puppies too young to travel legally (Dogs Trust 2014). In some circumstances, local ‘legitimate’ (licensed or accredited) breeders may be ‘laundering dogs’ through their facilities from third parties. Instances where puppies advertised in Scottish locations were being bred out of the country but sold by third-party locals have been reported (Maher & Wyatt 2019). Meanwhile, the ‘fragmentation’ of the market allows degrees of separation between buyer and breeder, trader and seller, making tracing almost impossible. Consumers report being reassured when visiting prospective puppies in locations that are ‘ticking’ the safety boxes of the Puppy Contract (SSPCA 2017). Post-purchase, temporary addresses and phone numbers supplied by the traders are quickly abandoned to further reduce traceability (Maher 2017). Although much effort has been expended to help bring the issue to light and suggest policy reform, Maher reports “*insufficient commitment and clarity*” in these attempts.

### Tracking and tracing

In comparison to other species, such as cattle and horses, the traceability of dogs in the UK and Europe is very limited (Table 2) (DEFRA 2023). Under current UK regulation, a cow must be traced from birth, throughout every change of location and ownership, alongside key health checks and veterinary treatments until its death (UK Government 2024b). This history can be accessed from the Centralised Cattle Tracing System (CTS) for ten years after the cow’s death. Inversely, the only form of identification a dog must carry is a microchip implanted subcutaneously, which only stores a unique ID number (UK Government 2022). This must be inputted into one of 14 decentralised databases, where only voluntarily provided owner information is recorded. A report by the Battersea Dog and Cat Home (2023) revealed that 72% of stray dogs collected have an inaccurate record on a database. Although microchip databases may provide the microchip’s country of origin, this does not necessarily correlate with that of the dog, as evidenced from puppies seized in 2022 at the Scottish border in Cairn Ryan where puppy farmers in eastern Europe had inserted Irish microchips into animals found to have been bred in Hungary (SSPCA 2024).

**Table 2.** A comparative table of the DEFRA guidelines to mandatory bovine, equine and canine identification and tracing practices in the UK (DEFRA 2023; UK Government 2024)

Mandatory Bovine, Equine and Canine Identification and Tracing practices in the UK.			
	Cows	Horses	Dogs
Identification	Ear Tags (visual and electronic [EID]) attached at 20 days old. Ear tag displays unique number and country of origin, without need of a scanner.	Equine Microchip (by 12 months old or before they are sold or moved). Equine passport issued by an approved Passport Issuing Organisations (PIOs) includes microchip number, physical description, age and movement history.	Dogs microchipped subcutaneously at 8 weeks of age. Microchips hold owner supplied information such as address, phone number, and pet information such as age and breed. Microchips can only supply information when scanned and chip number entered correct database (not available to public/owners, only available to officials and vet practices with affiliations to specific database).
Tracing	Centralised Cattle Tracing System (CTS) managed by APHA, tracks cows from birth to slaughter. EIDs are scanned prior and post movement, and all movement information is input into the system. A historical log of a cow’s movement is retained by CTS for 10 years after death.	Passport tracks all movements (ownership, sales, import), vaccinations and health states of the horse. Passport and historical passports must be retained until death of the horse.	Privately run databases hold microchip information. Information must be updated voluntarily by owners. No owner history, health information or movement history is retained by these databases.
Import	MUST be tagged and registered with CTS on arrival in UK. Must pass UK health and safety standards via veterinary health inspections- upon arrival. Must hold health certificates confirming they are free of disease (e.g. Bovine Spongiform Encephalitis and Foot and Mouth Disease.)	Must be microchipped. Must hold passport. Must pass UK health and safety standards via veterinary health inspections- upon arrival. Must hold health certificate stating horse is free of disease (e.g. equine flu, equine herpesvirus).	Must be microchipped. Must be vaccinated for rabies Must hold a pet passport. Must hold tapeworm treatment certificate (usually in passport) May need a health certificate depending on country of origin.
Movement	To move cattle, a license must be held. CTS tracks and stores all movement data from birth to slaughter.	Passport records all movements to include sales, transfers, changes of owner and import/exports. Transport for breeding, racing and slaughter requires documentation to confirm health status.	No requirement for movement licences or tracking. Microchip holds owner data as and when it is supplied. This can be checked by scanning the microchip and contacting database that holds that microchip. Database name is not supplied in microchip scan data.
Ownership verification	CTS tracks and stores current and historical ownership data, updated at each transfer.	Passports hold current and historical ownership data.	Microchip stores one set of ownership data (as supplied by owner). This is not necessarily the current owner, and no historical ownership data is stored. Microchip does not serve as proof of legal ownership. If a microchip is scanned and owner data supplied, a vet or rescue centre may contact the owner.
Health and disease monitoring	In case of disease outbreak, a cow can be traced back through all channels, to its origin.	Passport supplies information from first movement/sale to death and serves as a disease tracing document in case of diagnosis or outbreak.	No routine health tracking.

### Legislation, regulation and intervention

#### Education and intervention

For Christmas 2019, the Dogs Trust ran a puppy-farming awareness campaign, released via local government social media accounts and websites (Dogs Trust 2019; Trading Standards UK 2023) and several high-profile investigative documentaries followed (Poling S & BBC Scotland 2015). In response to the growing concerns associated with the UK puppy trade, the Dog Breeding Stakeholder group was formed in 2008 (by DEFRA, Dogs Trust, RSPCA, APGAW, Blue Cross, AWF, BVA, The KC and PDSA) ultimately leading to the Puppy Contract, developed to support buyers and sellers to breed and buy puppies responsibly under a mutual agreement (AWF *et al.* 2008). The use of a contract — though not necessarily the Puppy Contract itself — is identified as a requirement for achieving the higher standards set out in the statutory guidance for dog breeding under the LAIAR England (2018); Scotland (2021) (UK Government 2024a). These contracts provide a voluntary framework intended to promote responsible breeding and purchasing, but there is currently no empirical evidence as to their application or effectiveness. In Scotland, both the SSPCA Assured Breeder Scheme (SSPCA 2018), a voluntary accreditation programme that promotes higher welfare standards among responsible dog breeders, as well as the Codes of Practice outlined by the Welfare of Dogs (Scotland) Act 2025, provide guidelines for a good standard of welfare, but their efficacy depends largely upon consumer engagement and breeder good faith, particularly when purchasing a puppy online, where anonymity and limited face-to-face interaction is known to facilitate fraud, misrepresentation, and identity deception (Edwards *et al.* 2024).

#### Online regulation

The Electronic Commerce Regulations 2023 (post-Brexit replacement for a former EC Directive) requires some level of regulation on online advertising sites, characterised as an ‘information society service’. The LAIAR mandate that all adverts must contain clear, accurate, and comprehensive information to ensure transparency and protect animal welfare. Specifically, adverts are required to disclose the breeder’s or seller’s identity, including their registration or licence number where applicable, the breed, age, and sex of the animal, as well as health status and vaccination records. Additionally, details concerning the animal’s living conditions, lineage or pedigree, and any behavioural or special needs must be provided. However, no formal enforcement protocols mean platforms rely upon self-monitoring (The Electronic Commerce Regulations 2003).

The PAAG, formed in 2001, collaborates with animal welfare organisations, trade associations and veterinary bodies (with support from the UK Government) with the aim of regulating the online pet market (PAAG 2016). It has outlined guidelines representing ‘minimum standards’ for collaborating websites that advertise domestic animals for sale. Websites ‘agree’ to enforce these, advertising their collaboration with PAAG as a marker of ‘good practice’ (PAAG 2016). Despite being a force for good, PAAG collaboration is voluntary, and does not ensure that dogs sold on collaborating websites are not being traded unethically or even unlawfully.

### Legislation and regulation

Almost a decade has passed since a 2016 DEFRA consultation concluded that legislation governing the UK puppy trade was “*outdated, inflexible, incompatible with current welfare legislation and cumbersome for both enforcers and businesses*” (Maher 2017). Key issues raised in the consultation were inconsistent enforcement, lack of resource allocation, little to no random and/or unannounced visual inspections, lack of regulation of online trade and limited strategy to reduce consumer impulse buying.

A broader and systemic issue regarding the way in which animal welfare law is developed means legislation in the field is often restricted by characteristic drafting limitations and a lack of ‘future-proofing’ (Jones 2010). These issues, evident when reviewing the movement of bills through parliament, constrain legislative capacity to respond effectively to evolving practices and emerging challenges, especially in light of the rapid growth of internet marketplaces for live animals. This may be attributable, in part, to a consistently low political prominence of animal welfare as a policy area as reported by the UK Centre of Animal Law (A-LAW), which often results in insufficient prioritisation of the development of comprehensive and practicable legislative and regulatory frameworks, despite stakeholder efforts (A-LAW 2021). There is also perhaps the tendency to dismiss animal welfare as a peripheral or ‘soft’ concern despite the fact that, as demonstrated by this review, animal welfare patently engages a critical intersection of environmental, public, safety and ethical issues.

As animal welfare legislation in the UK is a devolved matter, each devolved nation is responsible for their own legal and regulatory framework. These include the Animal Welfare Act 2006 in England and Wales, the Animal Health and Welfare (Scotland) Act 2006, and the Welfare of Animals Act (Northern Ireland) 2011. All have established that causing unnecessary suffering to an animal is an offence (AWA/WAA Section 4) and assigned a legal ‘duty of care’ to those responsible for an animal’s care (AWA/WAA Section 9). As such, breeders that directly compromise breeding stock and puppy welfare by way of negligence, or intentionally in order to increase profit-margins, may be prosecutable. However, it is inherently complex to establish actionable causation in certain cases, for example, cases of the intentional breeding of two severely brachycephalic dogs to knowingly produce offspring to similar or more exaggerated conformation, leading to parturition difficulty and emergency C-section. In these cases, it can be difficult to distinguish between a proximate consequence of intentional selection for exaggerated cranial morphology and mere physiological coincidence. As this remains uncertain, it makes it difficult to ascribe legal liability for any increased incidence of surgical intervention and/or any associated harm to the breeding mother and her puppies.

Until its repeal in 2018 (England) and 2021 (Scotland), the Breeding and Sale of Dogs (Welfare) Act 1999 permitted inbreeding as a method of positive selection, a provision that remains under it’s successor: The Licensing of Activities Involving Animals Regulations (LAIAR). Canine geneticist Dr. Mark J. Neff (2014) suggested that genetic issues may have progressively accumulated over approximately 150 years since the formal classification of these breeds (Neff *et al*. 1999). It could be theorised that these issues may have subsequently been accelerated by the intensified demand for specific breeds of puppy, after the trade’s move online both within and outwith pedigree lines. As the evidence showcasing the detrimental effects of extreme conformation and genetic bottlenecking within the dog populations grows, it could be argued that ongoing regulatory acceptance of such breeding practices may be in conflict with the welfare standards set forth in the Animal Welfare Act (2006). However, the design of these acts does not currently extend protection to animals in their foetal or embryonic form. This exclusion breaks the ‘chain of causation’, making it difficult to establish criminal liability for harm caused to puppies produced by breeders engaging in irresponsible selective breeding. Official breed standards such as ‘long body’, ‘domed forehead’, ‘short legs’, or ‘wrinkled skin’ that may appear benign when qualified by ‘but not excessively’, are well-documented to be associated with health and welfare issues and to diminish an animal’s quality of life, so much so as to induce long-term suffering and premature fatality. Still, under current law, no prosecutable offence is committed by breed clubs that are not only promoting but incentivising the production of dogs to these standards. Likewise, A-LAW reports that no prosecutions can yet be brought against the intentional or unintentional breeding of dogs to such extreme conformation, or within such restricted gene pools, to knowingly or, negligently, cause their progeny to suffer (A-LAW 2021).

This issue in particular exemplifies how existing legislation, and even current approached to lawmaking, may be failing to keep pace with the evolving nature of issues influenced by the current online environment.

Whilst these acts outline the overarching principle of animal welfare and duty of care, regulations give detailed effect to the acts’ general provisions by prescribing specific standards, enforcement mechanisms and procedures for compliance (and penalties for non-compliance). The most pertinent regulation within the puppy trade is breeder licensing.

### Licensing

Under current regulations, breeders in the UK that breed over three litters annually are required to hold a licence, provision and enforcement of which is carried out by their local authorities. To receive a licence, a breeder must adhere to guidelines for record-keeping and animal welfare, subject to inspection, although there are no national guidelines as to how, when and by whom these checks are carried out to ensure compliance (UK Government 2021). Until 2021, breeders who had participated in the Kennel Club’s (KC) Assured Breeder Scheme (ABS) for three years or more were automatically granted a ‘five-star’ breeding licence valid for three years, exempting them from local authority inspections (Maher 2017). The scheme required adherence to animal welfare guidelines and included inspections to verify compliance. Historically, membership in the ABS contributed to a lower-risk designation under regulatory frameworks, as the scheme was accredited by the United Kingdom Accreditation Service (UKAS). In Scotland, the LAIAR operates as a risk-based regulatory regime, where the frequency and intensity of inspections are determined according to the assessed risk level of each licensed operator. Higher-risk businesses, evaluated based upon factors such as compliance history and conformity with recognised standards, are legally required to be subject to more frequent inspections. Meanwhile, “lower-risk” operators may be inspected less often. Compliance with UKAS-accredited standards remained a key metric in these risk assessments until 2024, when the Kennel Club allowed its UKAS accreditation to lapse and imposed a temporary freeze on ABS memberships pending a review of the scheme (The UK Kennel Club 2024). Consequently, local authorities should no longer consider ABS membership when assessing risk, but it is unclear whether this change is being applied consistently as of yet, raising concerns about potential inconsistencies in enforcement and the continued reliance upon outdated risk indicators.

Before the introduction of the LAIAR, English local authorities enforced legislation under the guidance of DEFRA, supported by informal networks, such as the Animal Welfare Licensing Forum (AWLF) and the National Companion Animal Focus Group (NCAFG). In Wales, enforcement is managed by local authorities with oversight from the Welsh Government’s Animal Welfare Team, and collaboration occurs through engagement with stakeholders, including RSPCA Cymru, albeit without a formalised working group. In Northern Ireland, responsibility is split between local authorities (for companion animals) and DAERA (for farmed animals, although farmed puppies do not come under this remit), with coordination occurring through regional enforcement groups. However, none of these structures provide the same formal, national platform for intelligence-sharing and policy feedback.

Local authorities (LAs) in England and Scotland are primarily required to assess compliance with the licensing conditions set out in Schedule 6 of the LAIAR, rather than relying solely upon risk-based assessments. In addition, LAs must adhere to the statutory guidance issued to support the effective enforcement of these regulations. LAIAR Regulation 10 further mandates that local authorities produce an inspector’s report following each inspection. While LAs retain discretion over the specific content of these reports, inspection templates reviewed to-date typically indicate a systematic approach, with inspectors evaluating compliance against each individual licence condition, in turn. This process should theoretically ensure a thorough and condition-specific assessment, reinforcing regulatory oversight and consistency in enforcement. However, discrepancies within inspection methods have been noted. Indeed, a report from Four Paws International (2025) highlighted the significant inconsistencies in the enforcement of breeder licence enforcement, UK-wide. While regulations require inspections for all new and renewal applications, FOI data suggested these are often not conducted, whilst licences were routinely renewed with minimal scrutiny, and very few were ever suspended or revoked — just 35 out of ~10,000 over the five-year data collection period (2018–2023). Authorities were reported to be issuing improvement notices instead of pursuing suspensions, revocations, or prosecutions due to limited resources and to low deterrent effect of current infraction penalties (Four Paws UK 2025). A 2025 report by Natalie Harney of the Naturewatch Foundation revealed that licensed breeders supply only 15-20% of the approximately one million dogs and puppies entering UK households annually, while around 6% are imported commercially, leaving the majority sourced from unknown, unlicensed, or potentially illegal origins (Harney, 2025).

### Legislative reform

Persistent lobbying by parliamentary committees and NGO stakeholders has led to several inquiries, most recently by EFRA in 2024, the Pet Welfare and Abuse enquiry prompted a call for expert evidence on the puppy trade.

Multi-stakeholder efforts have resulted in recent changes in legislation, including the aforementioned implementation of the LAIAR (UK Government 2018, 2021) which increased protection of animals bred and sold commercially by bringing stricter licensing of breeders and sellers, as well as the ban of third party sales (e.g. ‘Lucy’s Law’) which came into force through the UK in 2020, requiring all dogs under six months of age to be bought directly from the breeder, or re-homed from a rescue organisation (DEFRA and Rutley 2020).

Most recently, the Welfare of Dogs (Scotland) Act, originally introduced as a Private Members Bill in 2017, was finally passed in 2025 (Scottish Parliament 2025). The Act initially proposed measures such as mandatory registration of all puppy litters prior to sale or transfer, aiming to combat unethical breeding and puppy farming. It also placed significant emphasis on public awareness campaigns to educate prospective dog owners about their responsibilities. However, the Bill was substantially diluted during its passage through Parliament due to limited funding and challenges related to enforcement. The final version focused primarily on establishing minimum welfare standards for dogs, while many of the original regulatory provisions were scaled back. The Animal Welfare (Import of Dogs, Cats and Ferrets) Bill, sponsored by Dr Danny Chambers, MP is, as of April 2025, currently moving through parliament, in a bid to ban the import of puppies and kittens under six months, and dogs and cats that are mutilated or heavily pregnant (Chambers 2025).

### Animal welfare implications

The animal welfare implications of the online puppy trade are systemic and multi-faceted due to the evolution of an incipient, anonymous and profit-driven trade, enabled by a paucity of legislative reform. This review was written in order to bring to light the evidence available on these issues to support much-needed development of more evidence-based, effective and welfare-focused intervention and policy reform.

## Conclusion

This review provides a snapshot of the UK online puppy trade as it stands as of April 2025, identifying systemic issues associated with the current nature of the UK online puppy trade, including negative animal welfare, environmental and public health concerns and links to crime. Many of these issues can be associated with consumer culture, lack of transparency/traceability and outdated regulation/legislation.

A growing body of scientific literature is serving to bolster these reports and the ongoing efforts of stakeholders to ensure improvements within the industry and it’s governing policies. Ultimately, the evidence available suggests that the current laws that govern the breeding and trade of puppies in the UK can no longer protect the welfare of animals, or the rights of consumers. They are also failing to mitigate risks to public and environmental health or to prevent the trade from funding organised criminal activity. Further research into the underlying factors that drive this complex issue is essential in order to pursue an evidence-based, collaborative approach that may be effective enough to curtail the trade’s widespread negative impact.
